# The Relationship Between Non-elite Sporting Activity and Calcaneal Bone Density in Adolescents and Young Adults: A Narrative Systematic Review

**DOI:** 10.3389/fphys.2020.00167

**Published:** 2020-03-06

**Authors:** Hansa Patel, Luke Sammut, Hayley Denison, Paul Teesdale-Spittle, Elaine Dennison

**Affiliations:** ^1^School of Biological Sciences, Victoria University of Wellington, Wellington, New Zealand; ^2^Rheumatology, University Hospital Southampton, Foundation Trust, Southampton, United Kingdom; ^3^Centre for Public Health Research, Massey University, Wellington, New Zealand; ^4^MRC Lifecourse Epidemiology Unit, University of Southampton, Southampton, United Kingdom

**Keywords:** calcaneal quantitative ultrasound (cQUS), adolescent, sport, bone, systematic review

## Abstract

**Introduction:** Osteoporotic fractures represent a major public health burden. The risk of fragility fractures in late adulthood is strongly impacted by peak bone mass acquisition by the third decade. Weight-bearing sporting activity may be beneficial to peak bone mass accrual, but previous studies have focused on elite sporting activity and have used dual energy X-ray absorptiometry as a measure of bone density. The authors performed a narrative systematic review of individual sports (performed non-competitively or at local level) and calcaneal quantitative ultrasound (cQUS) bone measures in young people.

**Methods:** Multiple databases were systematically searched up until the 31st of March 2019. The authors included studies of participants' mean age (11–35 years), reporting any level of recreational sporting activity and cQUS measures as well as excluding elite/professional sporting physical activity. Studies (title and abstract) were screened independently by two reviewers, and a third reviewer resolved any discrepancies. STROBE guidelines were used to check the reporting of observational studies. The Newcastle–Ottawa Scale was used to assess the risk of bias of the studies included in the review. The systematic review was registered with the International Prospective Register of Systematic Reviews (PROSPERO).

**Results:** A search yielded 29,512 articles that considered relationships between bone density assessed by any technique and sporting activity. Duplicate and out of scope abstracts were removed. This left 424 papers that were screened by two reviewers; of these, six met the inclusion criteria, including assessment by cQUS. The authors identified papers where sports were considered, included soccer (football), swimming, cycling, gymnastics, dancing, badminton, basketball, fencing, wrestling, and judokas. Although study heterogeneity prohibited meta-analysis, all six included studies reported significant benefits of weight-bearing non-elite sports on cQUS outcomes.

**Conclusion:** Our study found beneficial effects of non-elite sports participation on cQUS in adolescence and young adulthood, although further work is now indicated.

## Introduction

Osteoporosis is a major international public health problem through its association with fragility fractures (Cole et al., 2008). Osteoporosis is often described as a disease that occurs when one becomes older, more often in females, and preventative methods thus often focus on older people (Tan et al., 2014). However, childhood and adolescence are critical periods of bone development; modifiable lifestyle behaviors have a major impact on the development of bones throughout life, and peak bone mass (PBM) is a major determinant of later fracture risk (Hernandez et al., 2003). Previous studies have suggested that physical activity (PA) and dietary calcium intake during childhood and adolescence play a critical, synergistic role (Weaver et al., 2016). There are, however, a limited number of studies that have looked at the impact of participation in individual non-elite sports on bone health at the calcaneum in young people, with most studies focusing on the effect of elite sporting activity or organized sports on bone health as assessed using ionizing methods, such as dual energy X-ray absorptiometry (DXA) (Tan et al., 2014).

A number of previous systematic reviews have considered the relationship between sporting activity and bone health in this age group, but they have studied associations between dual energy X-ray absorptiometry (DXA) and sporting activity. The effect of sporting activity varied according to sex; the skeletal sites and bone outcomes were measured as assessed by DXA and peripheral quantitative computed tomography (pQCT), which gives an estimate of volumetric bone density and other assessments of bone strength at relevant sites, including the calcaneus (Zulfarina et al., 2016). Tan et al.'s (2014) systematic review assessed PA and bone strength: the findings indicated that bone strength modifications due to PA were related to maturity level, sex, and study quality. A review by Hind and Burrows (2007) reported that weight-bearing exercise enhanced bone mineral accrual during early puberty, but it was unclear which form of exercise was the most beneficial. Meanwhile, Nikander's et al. (2010) systematic review of targeted exercise for optimizing bone strength throughout life supported the use of exercise to develop bone strength in children at weight-bearing sites.

Overall, previous studies that aimed to understand the relationship between sport in young people and bone health used ionizing imaging tools, such as DXA (Morris et al., 2000; Matthews et al., 2006; Deere et al., 2012; Ito et al., 2017; Júnior et al., 2017; McVeigh et al., 2019). There has been an increasing interest in the use of heel ultrasound as an alternative assessment of bone density, which also provides structural information of bones. Ultrasound technology is a non-invasive, widely available, low-cost, and portable tool that provides an assessment of bone density and quality at a readily accessible weight-bearing site with high trabecular bone content (GE Medical Systems Lunar, 2010). Ultrasound technology has been shown to be associated with fragility fractures in older adults (Krieg et al., 2008). In addition, Hans et al.'s study's suggests there is potential to use QUS when DXA is unavailable to assess bone health (Hans and Baim, 2017). The aim of this review was therefore to assess the relationship between non-elite sporting activity and bone density, as assessed by heel ultrasound in adolescents and young adults, through a systematic search and a narrative synthesis.

## Methods

The systematic review study protocol is registered with the International Prospective Register of Systematic Reviews under the Registration number CRD42018080101 (Centre for Reviews and Dissemination, 2009). The initial protocol described reviewing the association between non-elite sporting activity and bone density, with the latter being assessed using any bone measurement method. While the search and screening process adhered to this, the authors of the current paper only included studies that had assessed bone density through cQUS. This was a pragmatic decision based on the number of studies identified. The additional data retrieved will be used in a separate report on relationships between DXA and elite sporting activities.

An electronic search of PubMed/Medline, Proquest, AUSPORT, Ausport Med, and Medline (Ovid) proceeded until the 31st of March 2019 to source the relevant articles under review (see Table 1 for a summary of search strings used).

**Table 1:** Summary of search strings used.

(sport OR sport^*^ OR exercise OR exercis^*^ OR physical OR soccer OR football OR rugby OR athlet^*^ OR swimming OR tennis OR gym^*^ OR basketball OR “martial art” OR boxing OR cycling OR recreation OR cricket OR hockey OR Ball or golf OR badminton OR cycling OR wrestling)
AND
(bone AND health) OR (bone AND mass AND density) OR DXA OR DEXA OR BMD OR BMC OR SOS OR BUA OR SI OR (hip OR spine OR heel) AND ultrasound
AND
(adolescent OR child OR girl OR boy OR juvenile OR teen^*^ OR young OR people OR student OR youth OR minor OR college OR school OR paed^*^ OR pedia^*^)

Include: Synonyms, related terms, opposites, international terms, alternative spellings, plurals, truncations and wildcards (^*^ or $ or # to substitute for one character within a word), and proximity operators NEAR, NEXT, ADJ.

Observational studies were the main type of study for inclusion; however, if baseline data could be extracted from trial/interventional studies, these were also included. Only full-text, peer-reviewed journal articles published in English, unless they could be translated fully using Google Translate, were included (Google, 2019). There were no limitations on sample size or country of origin.

### Participants, Interventions, and Comparators

The following search strategy was applied:

1. Exposure: Non-elite participation in sporting activity performed at school or leisure time as an organized or regular activity—either self-reported or measured objectively. Participation in any type of sporting physical activity (quantitative studies).
2. Outcome: Any bone heel ultrasound measures, such as speed of sound (SOS)/velocity of sound (VOS), broadband ultrasonic attenuation (BUA), stiffness index (SI)/quantitative ultrasound index (QUI).
3. Population

- Inclusions: The age of study participants was mean age 11–35 inclusive. Both sexes were included. Participation in named sporting activity at a local or regional level.
- Exclusions: Those with long-term disease or health issues, such as physical/mental disability, that directly affect bone health through treatment, supplementation, or medication were excluded. Animal studies were excluded. Any participation in competition(s) at an elite or national level was also considered an exclusion, although typically elite sport is considered sport participation at a higher levels, such as at division I and professional levels (Lorenz et al., 2013; Bellver et al., 2019).

Two independent reviewers (HP and LS) screened the abstracts and titles of relevant reports and articles in duplicate to determine whether these met the given criteria for inclusion in the systematic review. Any discrepancies were resolved through discussion or with a third reviewer (ED). Then, the reviewers independently screened the articles identified from the title and abstract screening to determine whether they met the inclusion criteria for the review, and a third reviewer's (ED) agreement was sought where appropriate. Where feasible, study authors were contacted by email for completeness and clarity. For those articles and reports meeting the inclusion criteria, their reference lists and bibliographies were screened for any additional relevant studies to be included in the systematic review.

### Methodological Assessment: Data Extraction and Presentation of Study Results

The review is reported using the guidelines Preferred Reporting Items for Systematic Reviews and Meta-Analysis (PRISMA) statement (Moher et al., 2009).

### Risk of Bias in the Included Studies

The STROBE (Strengthening the Reporting of Observational studies in Epidemiology) guidelines were used to check the reporting of observational studies (Vandenbroucke et al., 2007). The descriptive information of each study was extracted and summarized in Table 2. To assess the quality of the methods used in the selected studies, the Newcastle-Ottawa Scale (NOS) risk of bias assessment tool was used (Wells et al., 2019).

**Table 2:** Key study characteristics.

**Author/Country Setting**	**Type of study**	**Study size Population sport**	**Sports activity**	**Comparator/Controls**	**Bone measure & site**	**cQUS imaging tool**	**Key findings**
Vlachopoulos et al. (2018) England Sport Clubs and schools	Longitudinal (PRO-BONE study)	Total *n* = 116 Caucasian males	Sport (swimming, soccer, cycling) duration >3 years	Controls: no sport like soccer, swimming or cycling for more 3 h/week nor 3 years prior	QUS heel mean of both feet, measured twice	Lunar Achilles Insight (TM Insight GE Healthcare), Milwaukee, WI, USA).	12 months football participation associated better SI than for cycling or swimming
		Aged 13.1 ± 0.1	Actual average years of training ranged from 3.9 to 5.9 years		SI only		
		*n* = 37 footballers	Actual average hours of training per week ranged from 5.2 to 9.4 h				
		*n* = 37 swimmers	Actual average MVPA(min/day) ranged from 85.0 to 119.8	Actual average MVPA(min/day) ~83.2			
		*n* = 28 cyclists					
		*n* = 14 active controls					
Gomez-Bruton Spain Clubs and high schools	Cross-sectional study within a larger randomized controlled trial	Total *n* = 129 Caucasian males & females	Sport (swimming) duration >3 years, minimum of 6 h/week	Controls: normo-active with no participation in sports like swimming or aquatics regularly and no sporting activities more 3 h/week	QUS heel (non-dominant)	Lunar Achilles Insight (Achilles Insight, GE Health- care), Diegem, Belgium	cQUS results showed no significant differences between swimmers and controls;
		Aged 11–18	Competing in regional tournaments		SI, SOS, BUA		
		*n* = 77 swimmers (34 females/43 males)					
		*n* = 52 normoactive controls (23 females/29 males)					
Madic et al. Serbia Schools	Observational	Total *n* = 62 male soccer players	Sport duration >1 year	Control 90 min of PA/week at school	Both heels QUS	Sahara (Hologic, Inc., MA, USA) sonometer	Higher BUA and SOS Soccer players than controls
		Aged 10–12			SOS Left and right		
		*n* = 32 soccer	Actual average hours of training per week ranged from 10 to 15 h		BUA Left and right		
		*n* = 30 control regular school PA					
Yung et al. China Local university students	Cross sectional study	Total *n* = 55 Chinese male university students	Sport (swimming, dancing, soccer) duration >2 years; at least twice week for at least 2 h	Control no exercise (sedentary control)	QUS heel dominant and non-dominant heel measured, analysis on dominant heel	Paris, Norland Medical System, Fort Atkinson, WI, USA	All QUS parameters showed a significant linear increasing with the weight bearing and high impact exercise
		Aged 18–22			VOS, BUA, SI		BUA, VOS, SI Soccer players > dancers > swimmers > sedentary control group
		*n* = 15 soccer					
		*n* = 10 dancing					
		*n* = 15 swimming					
		n = 15 no exercise/sedentary control group					
Mentzel et al. Germany Regional sports schools	Cross-sectional study	Total *n* = 177 sportspeople, of which three participants excluded because of lower limb fracture	Sport duration undetermined; two training sessions/week of at least 90 min	Reference population used (age, size, and gender related)	Both heel (mean) QUS	Sahara (Hologic, Inc., Waltham, MA, USA) sonar	
		Aged 11–18 (*n* = 121 boys; *n* = 56 girls)			SOS (SDS) and BUA (SDS)		
		*n* = 43 athletes					For the level of activity: significant correlation to BUA only judokas and wrestlers
		*n* = 38 soccer players					For training sessions: SOS low negative correlation and BUA-positive correlation
		*n* = 12 badminton players					
		*n* = 7 basketball players					
		*n* = 8 gymnastics					
		*n* = 18 fencers					
		*n* = 16 wrestlers					
		*n* = 29 Judokas players					
		*n* = 1 tennis, *n* = 1 triathlon, *n* =1 weight training					
Nurmi-Lawton England Clubs	Mixed longitudinal 3 years/cross-sectional for mothers	Total *n* = 97 females	Sport duration average for 6 years, two or more 90-min training sessions weekly; trained >10 h/week; competed at club or regional level	Normo-active sedentary controls including walking to school and attended school PE classes	QUS heel	Contact Ultra- Sound Bone Analyser (CUBA; McCue Ultrasonic Ltd., Winchester, UK)	Gymnasts had up to 24–51% higher BMC and 13–28% higher BMD, depending on skeletal site than controls.
		Age Baseline 8–17 years of age			Mean of both feet, measured twice		
		*n* = 45 gymnasts		No sports training requiring year-round training; included two competitive swimmers as they were engaged in an activity the authors considered non-weight-bearing			
		*n* = 52 controls					

## Results

### Study Selection

**Figure 1:**
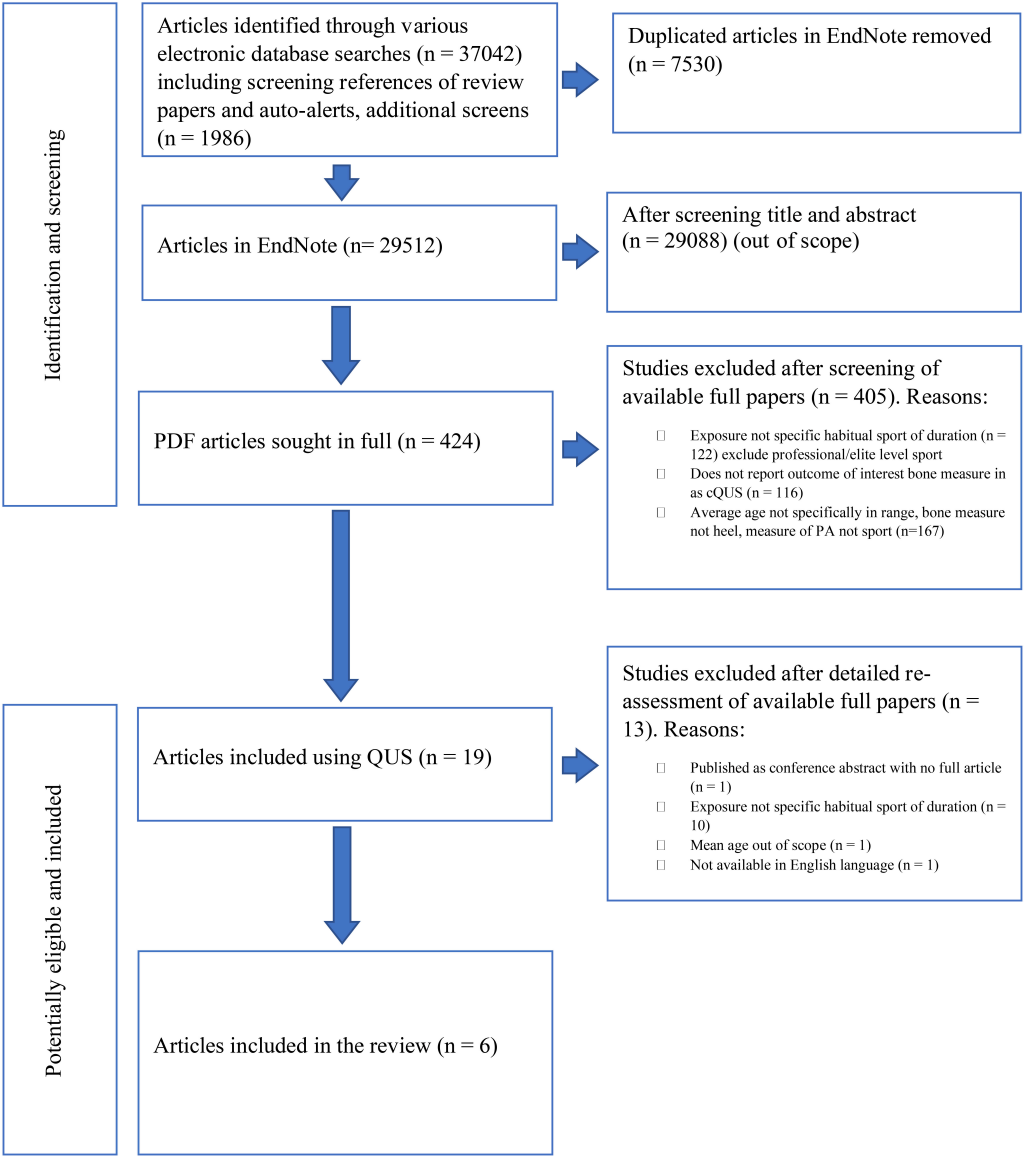
Flowchart of the literature search and the study selection process.

Figure 1 shows a flowchart of the literature search and the study selection process. The search yielded a reference list of a total of 37,042 articles. Duplicates were removed, leaving 29,512 articles to be screened by two independent reviewers. Based on the titles and abstracts, 29,090 articles were excluded, primarily because they did not use cQUS as a measure of assessment of bone outcomes. This left 424 papers to be assessed in full where available; the reviewers were unable to obtain the full manuscript for the study performed by Coaccioli et al. (2013) despite attempts that included a direct approach to the authors. In addition, the reviewers were unable to obtain a full translation from Chinese of a study by Qian (2017). As such, those two studies were excluded. Following full text screening, a total of six studies remained as meeting the inclusion criteria for this systematic review. The sports identified from this process were soccer, swimming, cycling, gymnastics, dancing, badminton, basketball, fencing, wrestling, and judokas (see Table 2).

### Risk of Bias Assessment

Using the NOS risk of bias assessment tool (Wells et al., 2019), the risk of bias of the six articles included in this systematic review was generally assessed as low to moderate, with one of the studies assessed as having a high risk of bias (see Table 3). The main area of bias identified was in the recruitment process of the studies; most studies failed to clearly indicate how the sample size calculation of the study was approached and how and why participants were invited to participate in the study. Some articles failed to report how many participants were approached to participate in the studies and why they were selected or not screened for the studies.

**Table 3:** Risk of bias (NOS).

**NOS tool**	**Yung et al**.	**Nurmi-Lawton et al**.	**Gomez-Bruton et al**.	**Mentzel et al**.	**Madic et al**.	**Vlachopulos et al**.
How well-described is recruitment of the exposed group?	Chinese University students—numbers approached not stated	Gymnasts recruited from five clubs—numbers approached not stated	Source of recruits was local swimming clubs and numbers approached/recruited provided	Recruited from College of Physical education—numbers approached not stated	Unclear—numbers approached not stated	Provided in separate referenced article; sports recruited were swimming/ football/cycling. Recruits came from sports club and schools
How were the exposed group selected?	At least 4 h sport each week for at least 2 years; different sports described	At least 10 h per week and competing in competitions.	Swimmers training for at least 3 years, training for a minimum of 6 h per week. Group subdivided according to whether participants were also training in another sport	At least 90 min per week	Soccer training for 10–15 h weekly for at least 1 year	Training for over 3 h per week for 3 or more years. Level of training provided for cases
How well-described is recruitment of the control group?	Chinese University students	Local schools; taking part in PE lessons only though 2 were competitive swimmers	Source of recruits was local schools and numbers approached/ recruited provided. Could not be doing any sport for more than 3 h per week	Used local reference data—exposure to sport in this group was unclear	“Not engaged in active sport.” Other details not provided	Provided in separate referenced article
Length of exposure to sporting activity	Variable between duration and time/week in different sports. Typically 2–3 years, range 7–15 h per week	Training for range of 2–12 years; average 6.5 years	At least 3 years	Unclear	At least 1 year	Range 4–6 years
Information on important confounders	Provided	Provided	Provided	Unclear	Unclear	Provided
Overall risk of bias	Moderate	Low	Low	High	Moderate	Low

The bias assessment tool indicated that three of the included studies (Gomez-Bruton et al., Vlachopoulos et al., and Nurmi-Lawton et al.) had low bias with clear study designs. Additionally, confounding factors, such as diet and PA, were acknowledged, and a detailed dietary assessment was available, and face to face interviews for PA were also carried out (Nurmi-Lawton et al., 2004; Gomez-Bruton et al., 2015; Vlachopoulos et al., 2018). Of the studies with moderate bias, Yung et al.'s study had small sample numbers in each of the four groups (*n* = 15) as well as limited details of recruitment (Yung et al., 2005). Yung et al.'s study performed a questionnaire that assessed the PA and diet of participants, and Madic et al.'s study was also assessed as moderate bias due the limitations of recruitment details and lack of dietary and PA assessment. Meanwhile, Mentzel et al.'s study was assessed as high bias due to reduced clarity of the level and intensity participants' different sports activities (Mentzel et al., 2005; Yung et al., 2005; Madic et al., 2010).

### Study Designs and Participant Characteristics

The studies extracted were too heterogeneous to allow for meta-analysis. A graphical display of the results and a summary of the key characteristics of the studies included in the review, along with a synthesis of the studies in a narrative form, is presented in Table 2. The sporting activity referred to in these studies included soccer, swimming, cycling, gymnastics, dancing and, to a limited degree, badminton, basketball, fencing, wrestling, and judokas.

Of note, the level of activity in the control groups was very different across the included studies. Vlachopoulos et al.'s study compared 116 young Caucasian male adolescents undertaking regular swimming, soccer, or cycling sports with active controls (including identifying participants that participated in other sports or swimming, soccer, or cycling for fewer than 3 h weekly) (Vlachopoulos et al., 2018). Gomez-Bruton et al.'s Spanish mixed gender cross-sectional study of 129 Caucasian children was part of a much larger controlled trial. The study compared the bone health of swimmers who competed at regional swimming tournaments at the start of the study with normally active control children who did other sports for fewer than 3 h a week and did not participate in other aquatic sports (Gomez-Bruton et al., 2015). In Madic et al.'s Serbian study of 62 participants, male soccer players were compared to controls who participated in regular school based sporting activity only (Madic et al., 2010). Yung et al.'s study of 55 Chinese male university students compared the bone effects of weight-bearing sports of swimmers, dancers, and soccer players by contrasting players with a sedentary control group of students who did not exercise (Yung et al., 2005). Mentzel et al.'s German study was a mixed study of 177 boys and girls from regional sports schools with various sports backgrounds whose bone health was compared against a reference population. The study presented limited details on how the levels of activity were assessed (Mentzel et al., 2005). Finally, Nurmi-Lawton et al.'s English study compared 97 female gymnasts and normally active controls (the controls did not participate in high impact sports for the past year at a competitive level, although two of the controls were competitive swimmers) (Nurmi-Lawton et al., 2004).

The six studies included in this review had a sample size that ranged from 55 to 177 participants. Five of the six studies included in this review studied school-aged children, with the study participants recruited from schools or sports clubs (Nurmi-Lawton et al., 2004; Mentzel et al., 2005; Madic et al., 2010; Gomez-Bruton et al., 2015; Vlachopoulos et al., 2018). The remaining study recruited students from their local university (Yung et al., 2005). The inclusion criteria varied amongst the six studies, with the main criteria being healthy children or adolescents with a reported sport history. General exclusion criteria in the studies included a history of chronic or musculoskeletal disease and taking medication that affected bone metabolism. Gomez-Bruton et al., Mentzel et al., and Vlachopoulos et al. specifically stated that participants with a known fracture history were excluded (Mentzel et al., 2005; Gomez-Bruton et al., 2015; Vlachopoulos et al., 2018). Mentzel et al.'s study also excluded children with a small shoe size participants that missed appointments (drop-outs), or participants that could not be located (Mentzel et al., 2005).

The mean age of participants in the studies included in this systematic review were between 11 and 22 years. Pubertal status was considered in the studies by Madic et al. (2010), Gomez-Bruton et al. (2015), and Vlachopoulos et al. (2018). None of the reviewed articles included the upper age range from 23 to 35. Collectively, 210 females and 426 males were included in this systematic review. One study included females only (Nurmi-Lawton et al., 2004), three studies included males only (Yung et al., 2005; Madic et al., 2010; Vlachopoulos et al., 2018), and two studies included both male and female participants (Mentzel et al., 2005; Gomez-Bruton et al., 2015). The ethnicity of the study participants was clearly stated in four of the six studies. Two studies recruited those who were of white healthy Caucasian ethnicity only (Gomez-Bruton et al., 2015; Vlachopoulos et al., 2018). The third study declared all the participants were white except for one participant in their study (Nurmi-Lawton et al., 2004), while the fourth study exclusively recruited Chinese university students (Yung et al., 2005). The Serbian and German studies did not state the study participants' ethnicity but, for the purposes of this review, the authors assumed their ethnicity based on each study's locality (Mentzel et al., 2005; Madic et al., 2010).

Nutrition was acknowledged as a factor of bone health in all six studies: four of the six studies completed some form of dietary analysis (Nurmi-Lawton et al., 2004; Yung et al., 2005; Gomez-Bruton et al., 2015; Vlachopoulos et al., 2018). A trained researcher helped participants complete a calcium frequency questionnaire in Gomez-Bruton et al.'s study (Gomez-Bruton et al., 2015). Yung's university students completed a 7-day recall for the participant's usual calcium intake (Yung et al., 2005). Similarly, Nurmi-Lawton et al.'s longitudinal study used regular estimated food diaries for the duration of this study (Nurmi-Lawton et al., 2004). Vlachoppoulos et al.'s study stated that one of its limitations was the lack of nutrition-related covariates in the analysis despite the fact that data was collected for the study (Vlachopoulos et al., 2018).

### Assessment Tool

The cQUS tools used included Lunar Achilles Insight (used in two studies), Sahara Hologic (used in two studies), Heel ultrasound densitometer Paris (Norland), Contact Ultrasound Bone Analyser, and Lunar Achilles Insight (TM Insight GE Healthcare, Milwaukee, WI, USA with OsteoReport PC (software version 5 GE Healthcare) (see Table 2). There was considerable variability in the bone measurements taken and the level of detail in the description of methods used to perform the measurements. All the papers employed statistical analysis using SPSS. The six study results were all presented *a priori* with *p* <0.05 being considered statistically significant, but, due to the heterogeneity of the tools and methods employed, output values were not directly comparable.

### Sports Participation—Duration and Intensity

The sports measured in this review included soccer, swimming, cycling, dancing, badminton, basketball, gymnastics, fencing, wrestling, and judokas. Lack of comparability of intensity of sports training and duration of involvement in regular sport made it hard to draw comparisons between studies. For example, in Vlachopoulos et al.'s study, athletic sports male participants at baseline had been engaged (≥3 h/week) in osteogenic (soccer) and/or non-osteogenic (swimming and cycling) sports for the previous 3 years or more (Vlachopoulos et al., 2018). Average years of training ranged from 3.9 to 5.9 years (Vlachopoulos et al., 2018). By contrast, Gomez-Bruton et al.'s study assessed swimming training in both girls and boys who had a previous history of swimming and competing in regional tournaments for more than 3 years and training for a minimum of 6 h per week (Gomez-Bruton et al., 2015). The inclusion criteria for this study was that participants had to have been training on a regular basis in a sport (cycling was not included) for more than 3 h per week for at least 3 years prior to the study. The swimmers were divided into those who were considered as pure swimmers, who had only participated in other sports for 1 or 2 years, and other swimmers, who were classified as participants in other sports for more than 2 h per week and/or other sports for a period of more than 2 years prior to the study (Gomez-Bruton et al., 2015). Madic et al.'s study on boys' soccer activity required that participants had a sport history of a minimum 1 year of active sports occupation in soccer with weekly training sessions of typically lasting up to 10–15 h (Madic et al., 2010). Yung et al.'s male university students were categorized by main sporting activity from high to low impact weight bearing and non-weight bearing exercises (soccer, dancing, swimming, and no exercise) (Yung et al., 2005). This exercise group of participants had to be engaged in supervised training in either soccer or dancing or swimming for at least 2 years twice a week for at least 2-h sessions (Yung et al., 2005). Mentzel et al.'s study with both boys and girls included eight sporting activities: soccer, badminton, basketball, gymnastics, fencing, wrestling, and judokas (as only one child each represented tennis, triathlon, and weight-training and, therefore, those sporting activities were not included in the analysis) (Mentzel et al., 2005). Sport participants had two or more 90-min training sessions weekly at the start of the study; past and current sporting activity details of participants were not stated (Mentzel et al., 2005). Finally, Nurmi-Lawton et al. studied female artistic gymnasts who had trained 2 or more years with more than 10 h of weekly training and had competed at club or regional level (Nurmi-Lawton et al., 2004).

The studies were not comparable for many reasons, including the duration and intensity of sporting activity of participants. For example, the athletes in Mentzel et al.'s study potential activity levels of the sporting participants could potentially be equated to Vlachopoulos et al.'s and Gomez-Bruton et al.'s control group (Mentzel et al., 2005; Gomez-Bruton et al., 2015; Vlachopoulos et al., 2018).

### Comparator (Control) Groups Activity Level

The details provided in the six studies for the comparator (controls) measurement for potential past sporting history and other physical activities (which may have impacted the bone measurements) was often lacking and was too heterogeneous to compare across the studies.

Vlachopoulos et al.'s study of athletic sports compared osteogenic soccer against non-osteogenic sports (swimming and cycling) with a small control group of 14 active boys who did not participate in any sports (soccer, swimming, or cycling) for more than 3 h per week or in the 3 years prior to the start of the study (Vlachopoulos et al., 2018). Gomez-Bruton et al.'s study of swimmers were compared with a control group who had neither performed in any aquatic sports on a regular basis nor participated in any other sport activity for more than 3 h a week (Gomez-Bruton et al., 2015). Madic et al.'s study on boys' soccer activity was compared to that of young boys not actively engaged in sport, aside from 90 min per week of PA at school (Madic et al., 2010). Yung et al.'s university male athletes were compared to a sedentary group who did not participate in exercise (Yung et al., 2005). In the study by Mentzel et al. (2005), athletes were compared to local reference data of 3,299 healthy Caucasian children and adolescents obtained from an earlier study by the same author. The two studies used the same conditions and same device, although details of the reference population's past sports history or PA level was not reported (Wunsche et al., 2000). Nurmi-Lawton et al.'s gymnasts were compared to controls who were involved in normal activities (including walking to school and physical education classes at school) for an average of 2.6 h weekly and not engaged in sports that required all year training at competition level (Nurmi-Lawton et al., 2004). The potential sporting activity levels of the control participants from Vlachopoulos et al.'s and Gomez-Bruton et al.'s studies, which reached a maximum of 3 h per week, may potentially equate to the sporting activity of Mentzel et al.'s participants, as this study included participants from a sports college that trained less than participants in other studies (Mentzel et al., 2005; Gomez-Bruton et al., 2015; Vlachopoulos et al., 2018).

### Bone Measurement Results

Overall, high-impact weight-bearing sports, such as soccer playing and gymnastics or dancing, were associated with the greatest benefits for bone health (Table 2). Swimmers and cyclists were not at any apparent bone advantage compared to controls. Hence Madic et al.'s study of male soccer players reported significant differences in cQUS between soccer players and controls (Madic et al., 2010). Yung et al.'s study found weight-bearing and high-impact exercise to be associated with higher QUS parameters. In particular, soccer players and dancers had significantly greater BUA, VOS, and SI than swimmers and the sedentary control group (Yung et al., 2005). In Vlachopoulos et al.'s study, soccer players had statistically greater cQUS ultrasound parameter SI compared to swimmers, cyclists, and controls at baseline (Vlachopoulos et al., 2018).

Similarly, Gomez-Bruton et al.'s gender study compared swimmers with controls and found no significant differences in any cQUS parameters when measuring the non-dominant calcaneus between any of the groups (Gomez-Bruton et al., 2015). In the only study to compare a very wide range of sporting activities, and with attendant power considerations for that reason, Mentzel et al.'s study showed significant differences between cQUS SOS and BUA between the sports students (a mixed gender study of 177 children aged 11–18) and the reference group. Although direct sporting comparisons were more challenging, the authors reported higher SOS values in athletes than wrestlers, in basketball players than fencers, in basketball players than wrestlers, and in gymnasts compared with judokas sports players (Mentzel et al., 2005).

Some studies investigated the level (or impact) of weight-bearing activity and bone health. While Mentzel et al.'s study did not observe strong correlations between increased weight-bearing activity in basketball (*n* = 7) and bone health, this may be reflective of the very small sample size and lower study power (Mentzel et al., 2005). As such, the authors consider that the results of Mentzel et al.'s study should be interpreted with caution (Mentzel et al., 2005). In Mentzel et al.'s study, judokas players and wrestlers showed a significant positive correlation between heel BUA vs. level of activity (Mentzel et al., 2005). Further, when considering age related SOS as an outcome, significant differences were shown between badminton players and gymnasts, between basketball players and fencers, as well as between judokas players and gymnasts (Mentzel et al., 2005). Finally, one study considered body build in more athletic young people; Numri-Lawton et al.'s study of gymnasts revealed that the gymnasts were smaller and lighter than controls, but they still had significantly higher QUSs (Nurmi-Lawton et al., 2004).

## Discussion

In contrast to many other reviews, this review focused only on specific non-elite sporting activity performed at a non-competitive level in young people as assessed by calcaneal ultrasound. The quality of the six articles was generally assessed as moderate quality, though variability was present, and methodological differences prevented a meta-analysis.

The overall aim of the six studies was to investigate the effects of different non-elite sporting activities, some of which were classified as weight-bearing and non-weight-bearing non-elite sporting activities of different intensities on bone mineral accrual in adolescence and early adulthood. The studies were heterogenous, but a consistent pattern emerged. Vlachopoulos et al. showed that boys playing soccer produced had better bone heel ultrasound outcomes than those who participated in cycling or swimming (Vlachopoulos et al., 2018). Similarly, Nurmi-Lawton et al. showed that female gymnasts had significantly higher bone density than controls (Nurmi-Lawton et al., 2004). Gomez-Bruton et al.'s study indicated there were no differences found in QUS parameters between swimmers and controls (both male and female) (Gomez-Bruton et al., 2015). Madic et al.'s study of male soccer players reported significant higher QUS values compared to controls (Madic et al., 2010). Mentzel et al.'s comparison of those children involved in sports found the QUS (SOS and BUA) parameters were significantly higher in sport participants engaged in weight-bearing activities compared to the reference data (Mentzel et al., 2005). Yung et al.'s study indicated a linear increase in all QUS measures as weight-bearing activity increased (Yung et al., 2005). In general, the six studies suggested that weight-bearing non-elite sporting activity was associated with higher QUS, and that some dose effect was reported with greater levels of sporting activity (frequency and duration).

Weight-bearing physical activity is thought to stimulate bone formation and thus improve bone mineral density (BMD) by exposing the skeleton to mechanical strain, provided that it is performed at a high enough frequency and high impact intensity (as evident in the studies that included swimmers or cyclists who had similar cQUS results to their comparative controls) (Yung et al., 2005; Gomez-Bruton et al., 2015; Vlachopoulos et al., 2018). Importantly, there is little epidemiological evidence that walking improves BMD (Martyn-St James and Carroll, 2008). Rather, mixed loading programs that included jogging, walking, and stair climbing consistently improve hip BMD in older people, although far fewer data exist in young adults (Martyn-St James and Carroll, 2009). The optimum type and level of PA for improving BMD remains unknown, and it is unclear whether a specific threshold strain needs to be exceeded. It is also unclear whether or not different loading movements in different sports may have varying effects on BMD and whether the effects are identifiable at different sites. Lower limb impact during weight bearing reflects their ground reaction force. In a study of adolescents from the Avon Longitudinal Study of Parents and Children, using pQCT and DXA found that vigorous PA (equivalent to jogging) was positively related to cortical bone mass, but no independent relationship was seen for moderate PA after adjusting for vigorous PA; this highlighted the importance of vigorous activity in this age group (Sayers et al., 2011). This also highlighted the importance of quantifying the intensity, frequency, and duration of PA in comparators controls when assessing the changes in cQUS measures associated with non-elite sporting activity.

There are several limitations to this systematic review. The QUS tools used varied, with distinct model versions used in the measurements undertaken. As such, the output values were not directly comparable. There was considerable variability in the bone measurements taken and the level of detail provided of methods used to perform the measurements. These methods varied from measuring both feet separately to find the mean of the two, performing measurements in duplicate or triplicate, performing measurements either on the dominant foot or the non-dominant foot, and measuring both left and right feet but presenting the results of the left foot only. Overall, the reproducibility of the QUS measurements within the individual studies themselves were within an acceptable range and researchers followed manufacturer's instructions validating the use of the QUS measurement. Unfortunately, two articles were not obtainable despite numerous attempts to search for the English translations of the full article or to contact the authors (Coaccioli et al., 2013; Qian, 2017). Funding precluded the use of an official translation service, and we were therefore reliant on Google Translate. This is a limited service; although the study by Mentzel et al. was subject to translation bias, its inclusion was justified as it was within the scope of this review. Mentzel et al.'s study was therefore translated from German to English using Google Translate, a freely available online tool. This translation may include inaccuracies since sentences could be translated out of context, especially when translating colloquial words or words with multiple meanings. Another limitation of this review is that the age of the study participants under review leaned toward the younger end of the 11–35 age group. The lack of detail regarding the power of the studies made it difficult to assess whether and how the sample size recruited affected the results. The ethnicity of the study participants was not always clearly stated in the study. Furthermore, although nutrition was acknowledged as a factor in bone health in all six studies, only four of the six studies completed any dietary analysis. Details for sports measurement for sporting history, duration, and other physical activities included were heterogeneous, and sometimes the methods of recording and confirming the details were ambiguous. For example, the duration of participation in regular sporting activity prior to enrolment in each study was often not provided. The mean weekly sport training regimes ranged from a minimum of 3 h to up to 27 h, and the level of participation in non-elite sporting activity between studies were not directly comparable. Inter-study comparisons of results may not be made as in the six selected studies the selection criteria for participants, and the controls were inconsistent between the studies. For example, some study participants selected for controls in one study would be sufficiently active to be participants in another study in this group of six studies. Finally, resource limitations meant we were unable to include SPORTDiscus and Web of Science in our search.

In comparison, a number of other systematic reviews have assessed bone health at other bone sites using various imaging tools, and this review complements those data. Our results are complementary and support the findings of those studies. Specifically, weight-bearing sporting activity, and particularly high-impact weight-bearing activity, appeared to be beneficial, while swimming did not enhance bone mineral accrual. Previous studies compared differing age ranges or assessed bone outcomes in relation to pubertal status, while some reviews have been undertaken in groups of young people participating in exercise regimes or individual sports such as swimming, soccer, gymnastics, and ballet, which may be at a combination of recreation or elite or competitive level. For example, in a systematic review undertaken by Nikander's et al. (2010), the authors found, using various imaging techniques, such as DXA, pQCT, MRI (Magnetic Resonance Imaging), and HSA (Hip Structural Analysis), that, in children, an exercise regime lasting more than 6 months enhanced bone strength at loaded sites, but this effect was not seen in adults. Gomez-Bruton et al. suggested that swimmers may not be reaching their PBM potential: Gomez-Bruton et al.'s systematic review in 2016 found higher DXA-derived BMD values in young Caucasian children and adolescents engaged in osteogenic sports relative to swimmers and controls (Gomez-Bruton et al., 2016). Gomez-Bruton et al.'s recent systematic review in 2018 focused on young adult swimmers aged 18 to 30 and found that, in these young adults, limited osteogenic effects of swimming during adolescence persisted through early adulthood (Gomez-Bruton et al., 2018). The systematic review by Lozano-Berges et al. (2018) also used various imaging tools and found children aged 6 to 18 playing soccer had positive bone mass outcomes compared to the controls (Lozano-Berges et al., 2018). Burt et al. (2013) systematically reviewed participation in gymnastics during the pre-pubertal growth period and found there was skeletal health benefits mostly for the upper body regions (Burt et al., 2013). Similarly, a systematic review by Wewege and Ward (2018) in pre-professional female ballet dancers found site-specific osteogenic effects compared to the controls. A systematic review by Krahenbühl et al. (2018) addressed the effects of weight-bearing sports, such as soccer and gymnastics, on bone geometry in children and adolescents, and found that the benefit was dependent on the frequency and intensity of the PA measured. The systematic review by Koedijk et al. (2017) assessed bone health in children up to the age of 24 and measured PA subjectively through questionnaires or objectively using an accelerometer, with a focus on sedentary behavior rather than a specific non-elite sport. Three of the studies identified by Koedijk et al.'s review that were of higher quality indicated that there was no association between sedentary behavior and total body bone outcomes as measured by DXA; although 12 of the studies included in the same review assessed the lower peripheral bone outcomes with DXA or QUS found a negative association with sedentary behaviors (Koedijk et al., 2017).

The authors chose to undertake this systematic review of non-elite sporting activity with bone health using cQUS as the outcome measure in order to capture studies that may not have been included in previous systematic reviews. The authors used a heel ultrasound as the outcome measure in this study to assess the effects of non-elite sports. Many studies indicated that a heel ultrasound is used to assess bone structure and strength and is used worldwide for osteoporotic fracture risk assessment when DXA the gold standard tool in diagnosis is not available, although it is not to be used as a diagnostic tool (ISCD, 2013; Quiros Roldan et al., 2017). The positive attributes of the heel ultrasound test are that it involves no risks or harm and is a cost-effective, comfortable, pain-free, and radiation-free test that is easy to use and only takes a few minutes to perform (Shewale et al., 2017; Komar et al., 2019). Studies have shown the quantitative ultrasound densitometry technique to be useful in assessing skeletal health status changes due to exercise in all age groups and as a research tool (GE Medical Systems Lunar, 2010; Babatunde and Forsyth, 2013; Yesil et al., 2013). Jaworski et al.'s study in 1995, Baroncelli's study in 2008, and Daly et al.'s study in 1997 found the use of ultrasound in normal healthy children to be a safe and non-invasive method when comparing the skeletal status of exercising children (Jaworski et al., 1995; Daly et al., 1997; Baroncelli, 2008). Different studies have used either the dominant heel, the non-dominant heel, or a mean of the two to measure the reported outcome. While it is possible that this might impact findings, the consistency of our results suggested this was not a major consideration here, particularly given the sports studied. Perhaps this plays a lesser role compared to the effect on the dominant limb in racquet sports such as tennis (Kontulainen et al., 2003).

## Conclusion

Although study heterogeneity prohibited meta-analysis, all six studies reviewed reported significant benefits of weight-bearing non-elite sporting activity in children and young adults. While both sexes were studied in several of these individual reports, small sample sizes made it difficult to dissect differences in outcomes between the two sexes. The studies revealed habitual levels of high-impact sports such as soccer produced better bone outcomes (particularly in males) compared to non-weight bearing sports such as swimming and cycling. Sporting behaviors commencing in the early years is an opportunity to improve PBM potential and set in place other healthy long-term lifestyle behaviors. More studies, especially in young adults in their 20s and 30s, are now urgently required to examine this issue in greater detail with more clearly defined control groups.

## Author Contributions

HP performed the searches. HP, LS, and ED reviewed the search results and extracted the data. HD provided advice and guidance regarding the systematic review methods. HP, ED, HD, PT-S, and LS edited the manuscript. All authors contributed to manuscript revision, read, and approved the submitted version.

### Conflict of Interest

ED has received consulting fees from Pfizer and UCB. The remaining authors declare that the research was conducted in the absence of any commercial or financial relationships that could be construed as a potential conflict of interest.
